# Supplementary material for: Transcriptomic–Proteomic Analysis Revealed the Regulatory Mechanism of Peanut in Response to Fusarium oxysporum
Source: Int J Mol Sci. 2024 Jan 3;25(1):619. doi: 10.3390/ijms25010619 (PMC10779420; doi:10.3390/ijms25010619)
Supplement: Supplementary file 1 [file ijms-25-00619-s001.zip › Supplemental Captions.pdf]

Supplemental Data

Figure S1. GO enrichment analysis of the DEGs. The vertical axis is DEGs enriched GO pathway and the horizontal axis is protein *p*-value in -log 10. (**A**) The pathway of upregulated DEPs enrichment. (**B**) The pathway of downregulated DEPs enrichment.

Figure S2. Sample clustering heatmaps and the PCA analysis of two omics data. (A) The clustering heatmap of the transcriptome data in six samples. (B) The clustering heatmap of the proteomics data in six samples. (C) The PCA analysis of the transcriptome data in six samples. (D) The PCA analysis of the proteomics data in six samples.

Figure S3. GO enrichment analysis of the DEGs and DEPs. The vertical axis is DEGs enriched GO pathway and the horizontal axis is protein *p*-value in -log 10. (**A**) Cluster heatmap of DEPs and DEGs enriched in Molecular function in six regulatory relationships. (**A**) Cluster heatmap of DEPs and DEGs enriched in Molecular function in six regulatory relationships. (**B**) Cluster heatmap of DEPs and DEGs enriched in Cellular component in six regulatory relationships. (**C**) Cluster heatmap of DEPs and DEGs enriched in Biological process in six regulatory relationships.

Table S1. The table of all DEGs

Table S2. The table of Go analysis of all DEGs

Table S3. The table KEGG analysis of all DEGs

Table S4. The detail of all differentially expressed proteins

Table S5. GO functional enrichment analysis of all DEPs

Table S6. KEGG enrichment analysis of all DEPs

Table S7. The network of DEPs

Table S8. Protein quantitation of all DEPs

Table S9. Protein and Transcript quantitative combination data

Table S10. GO enrichment analysis of DEGs and DEPs in two expression levels

Table S11. KEGG enrichment analysis of DEGs and DEPs in two expression levels
