# Supplementary figures and images for: Transcriptomic–Proteomic Analysis Revealed the Regulatory Mechanism of Peanut in Response to Fusarium oxysporum
Source: Int J Mol Sci. 2024 Jan 3;25(1):619. doi: 10.3390/ijms25010619 (PMC10779420; doi:10.3390/ijms25010619)

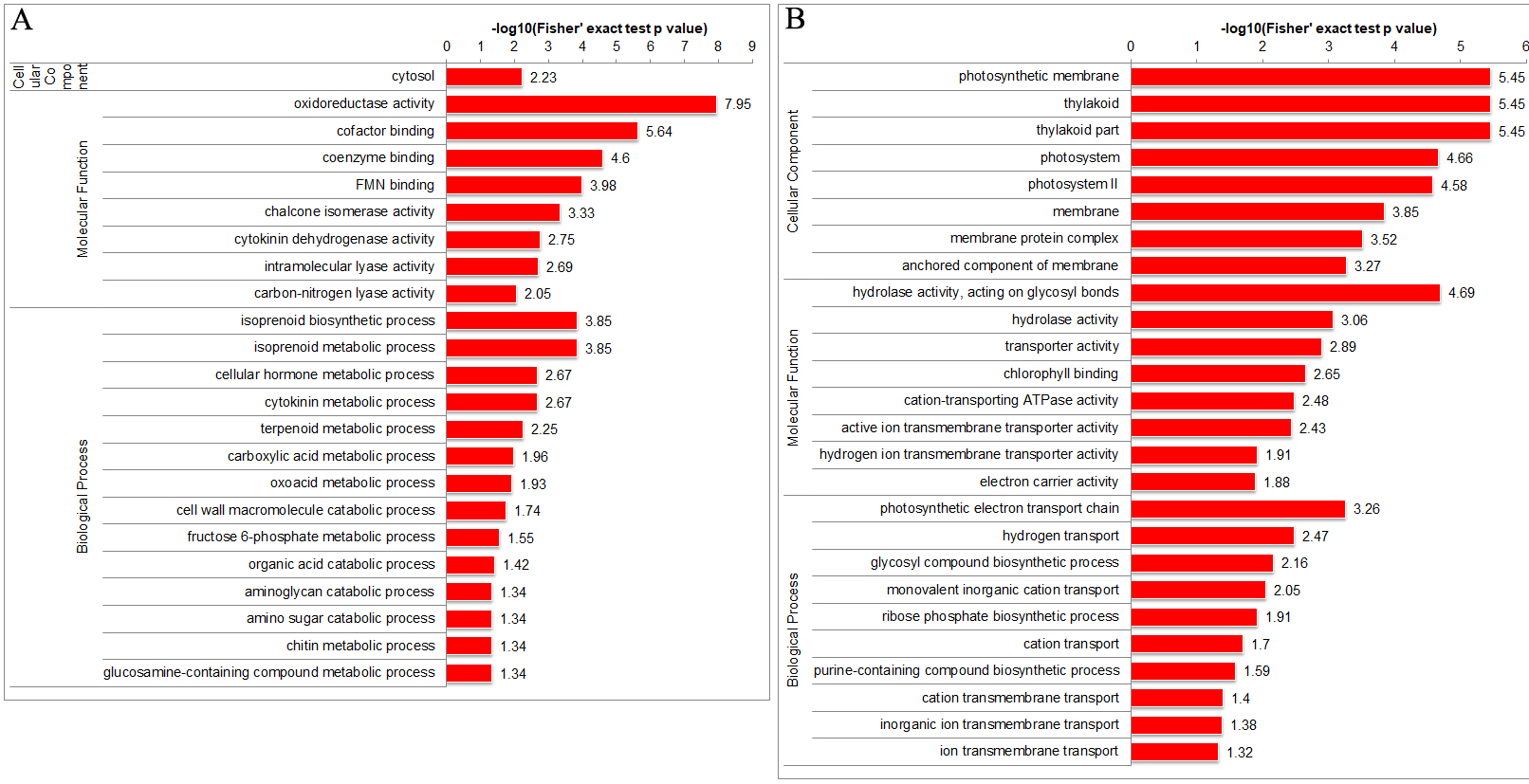

Supplement: Supplementary file 1 [file ijms-25-00619-s001.zip › Figure S1.jpg]

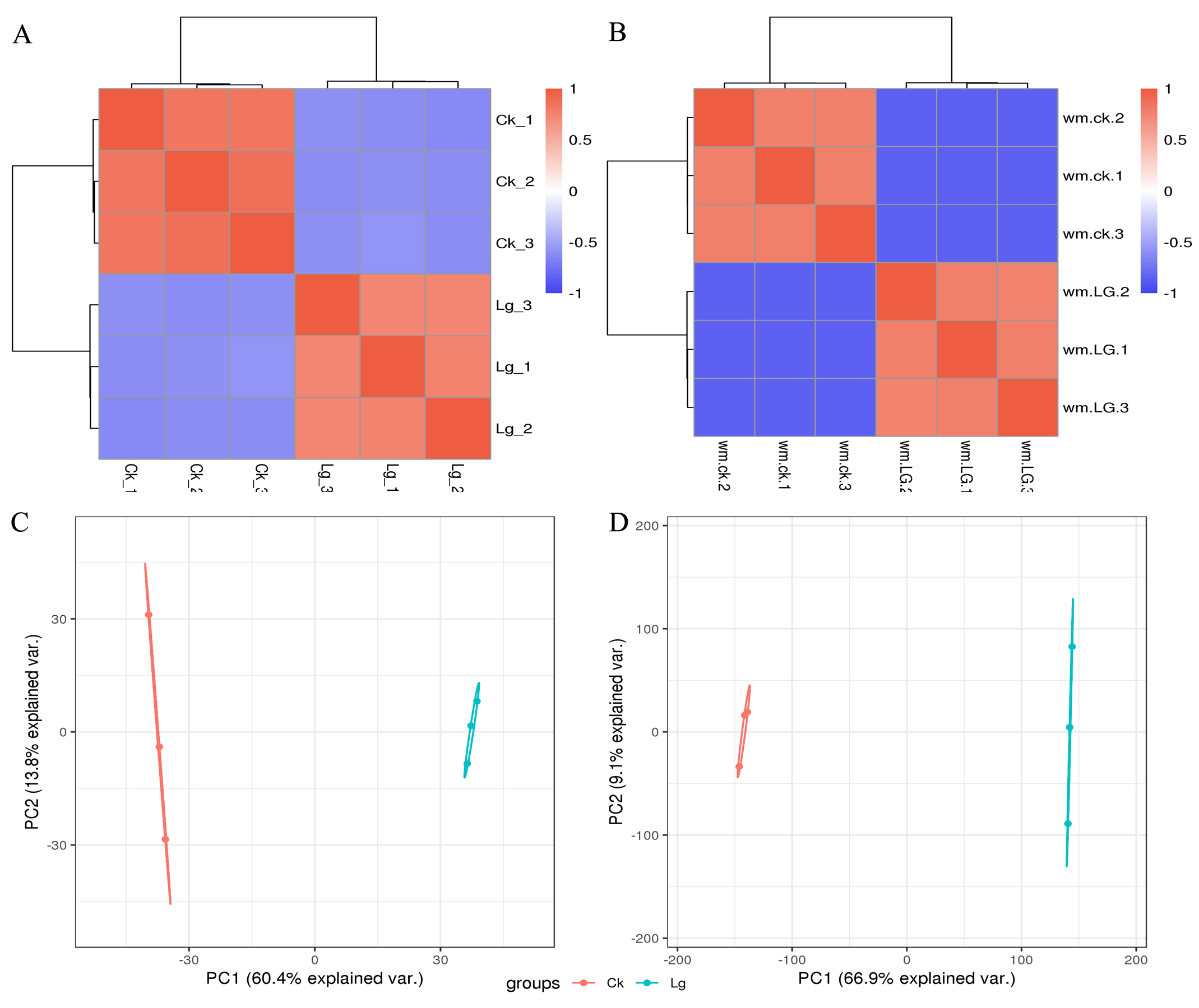

Supplement: Supplementary file 1 [file ijms-25-00619-s001.zip › Figure S2.jpg]

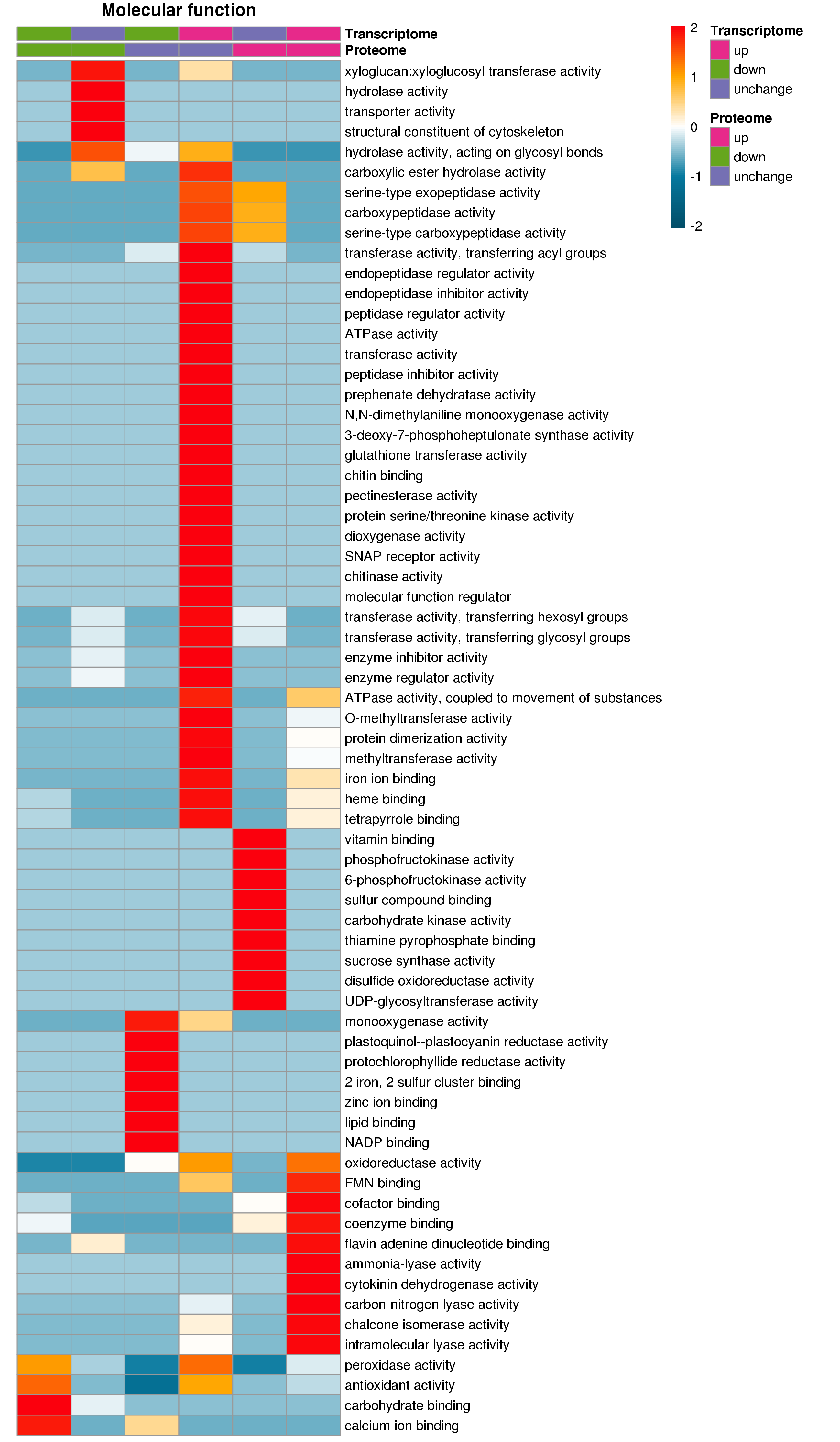

Supplement: Supplementary file 1 [file ijms-25-00619-s001.zip › Figure S3A.png]

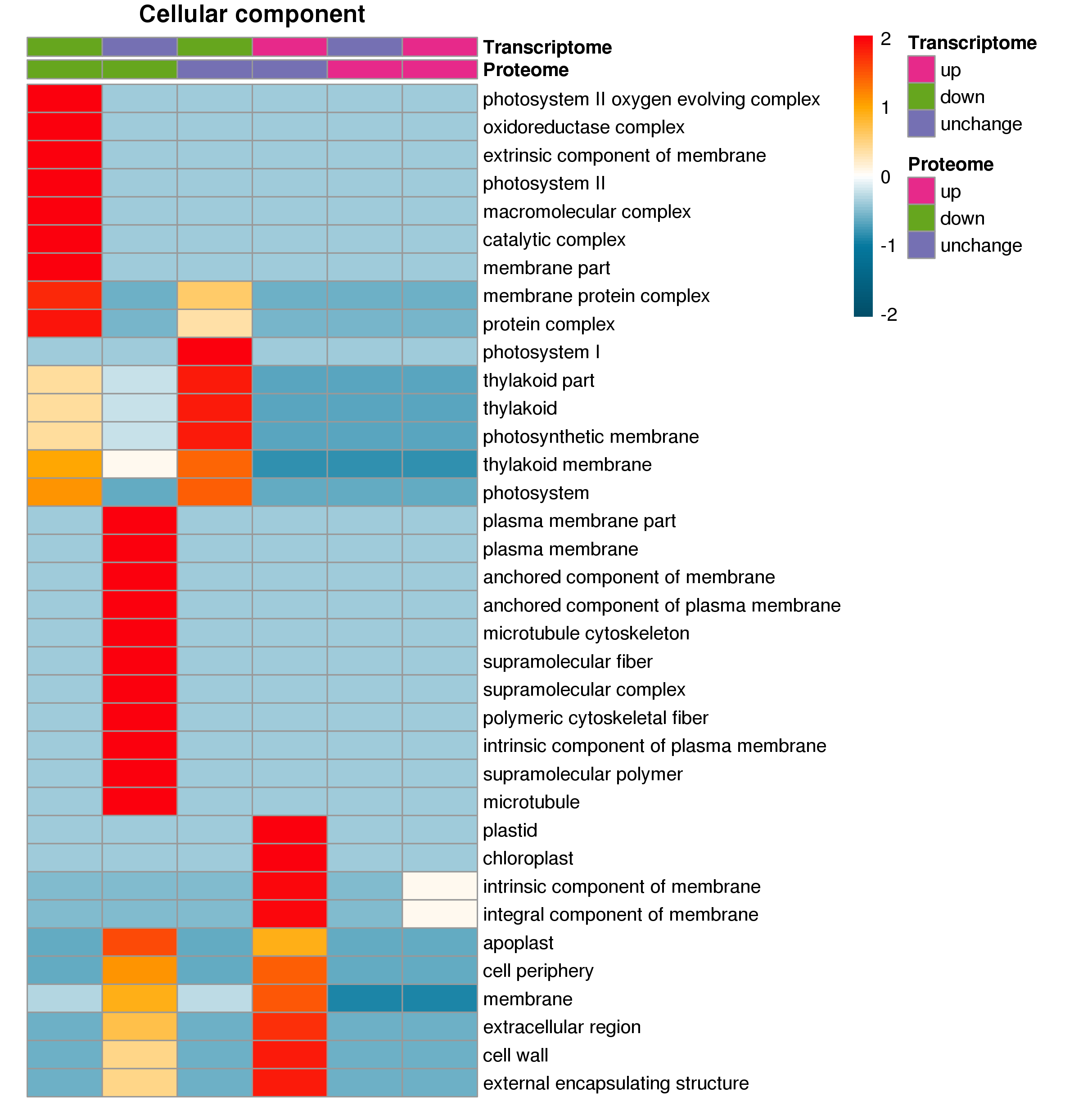

Supplement: Supplementary file 1 [file ijms-25-00619-s001.zip › Figure S3B.png]

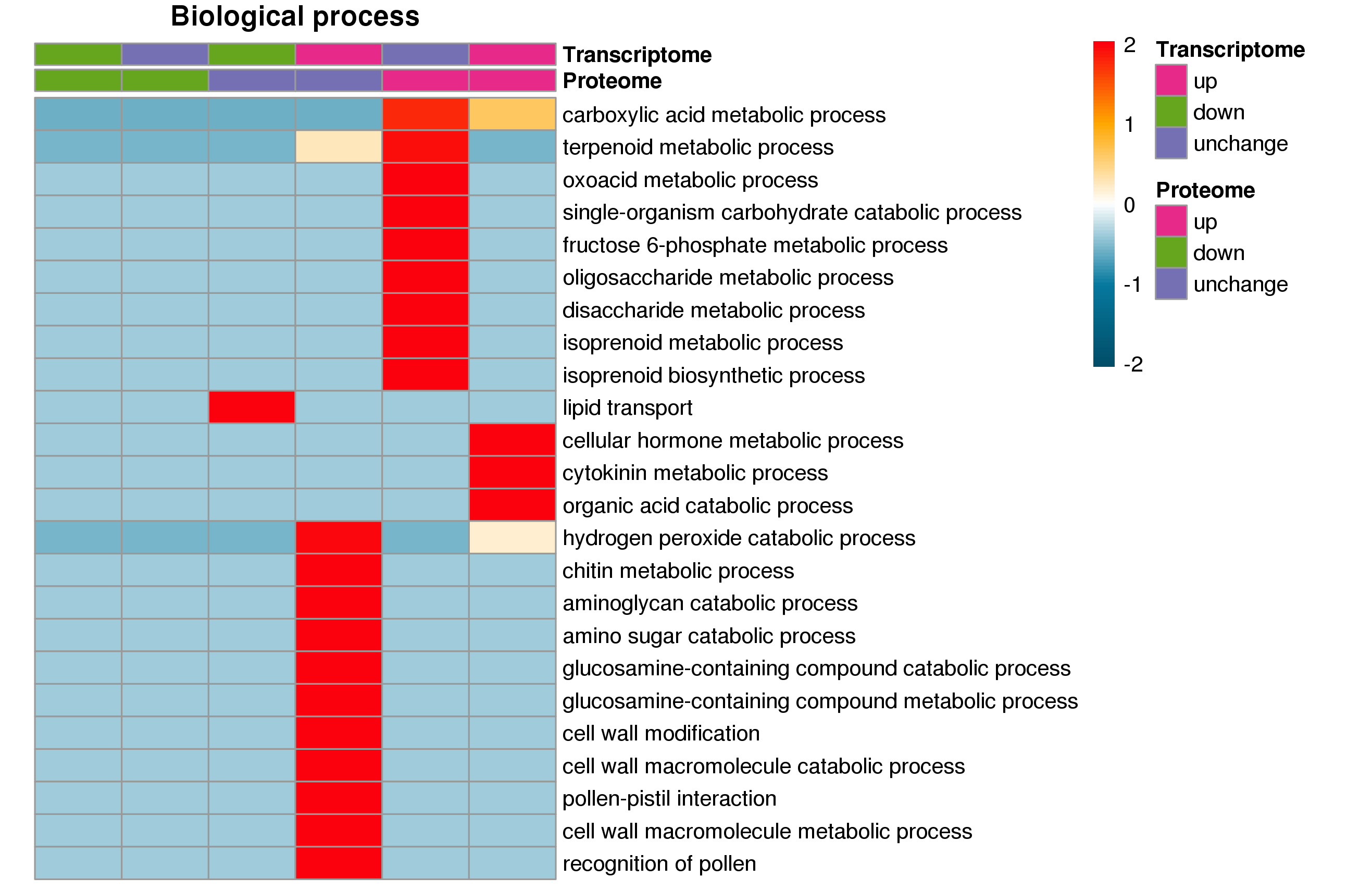

Supplement: Supplementary file 1 [file ijms-25-00619-s001.zip › Figure S3C.png]
